# Surgical Management of Gastric Gastrointestinal Stromal Tumor: A Single Center Experience

**DOI:** 10.4103/1319-3767.80382

**Published:** 2011

**Authors:** Ehab El-Hanafy, Mohamed El-Hemaly, Emad Hamdy, Ahmed Abd El-Raouf, Nabil Gad El-Hak, Ehab Atif

**Affiliations:** Gastroenterology Surgical Center, Mansoura University, Mansoura, Egypt

**Keywords:** Gastrectomy, gastrointestinal stromal tumor, wedge resection

## Abstract

**Background/Aim:**

Gastrointestinal stromal tumors (GISTs) are the most common mesenchymal tumors of the gastrointestinal tract. Surgery remains the mainstay of curative treatment. Our objective is to evaluate the outcome of surgical treatment of primary gastric GIST.

**Materials and Methods:**

Between January 1997 and April 2008, thirty seven consecutive patients underwent resection for GISTs (35 patients with primary gastric GISTs and two patients with intestinal GISTs who were excluded from the study). These patients underwent upper endoscopy ± biopsy, barium meal and abdominal CT scan. Patients’ demographics and clinical presentations were analyzed. Perioperative parameters measured included operative times, estimated blood loss, intraoperative finding, surgical techniques, morbidity and length of hospitalization. Recurrence and survival were also analyzed.

**Results:**

Of the 35 patients with gastric GISTs included in the study, 63% were female. The median age was 59 ± 14 years (range, 23 to 75 years). The primary presenting symptoms were bleeding and dyspepsia; 43% of these tumors were located mainly in the body of the stomach. Tumor size was < 10 cm in 80% of the patients. The average tumor size was 6.3 ±3.2 cm (range from 3 to 13 cm). Regarding the surgical management, 20 patients (57%) underwent gastric wedge resection, eight patients (23%) underwent partial gastrectomy and the remaining seven patients (20%) underwent total gastrectomy. Radical resections were found in 32 patients (91.5%) while palliative resections were found in three patients (8.5%). The resected lymph nodes were negative in 32 patients (91.5%). Recurrence was noted in three patients, with a median time to recurrence of 14.3 months (range, 7 to 28 months). The three- and five-years survival in patients who underwent wedge resection was 92% and 81%, respectively, where it was 95% and 87%, respectively, in patients who underwent gastrectomy (either partial or total). There were no major intraoperative complications or mortalities.

**Conclusion:**

Complete surgical resection either through wedge resection or gastrectomy with negative margins remains the gold standard treatment in the management of patients with primary resectable gastric GISTs.

Gastrointestinal stromal tumors (GISTs) represent a rare but distinct histopathological group of intestinal neoplasm of mesenchymal origin. Its incidence is only 0.2% of all gastrointestinal malignancies.[1] In the past, these tumors were believed to arise from smooth muscle; so they were classified as leimyomas, leimyoblastomas, and leimyosarcomas.[1,2] However, with the advent of electron microscopy and immunohistochemistry, a pleuro-potential intestinal pacemaker cell called the interstitial cell of Cajal was identified as the origin of GISTs.[3] This cell has myogenic and neurogenic architecture and is found within the myentric plexus, submucosa, and muscularis propria of the gastrointestinal (GI) tract.[3,4] GISTs are most commonly found in the stomach (40-70%), but can occur in all other parts of the GI tract, with 20 to 40 % of GISTs arising in the small intestine and 5 to 15% from the colon and rectum.[5] They typically grow endophytically, parallel to the bowel lumen, commonly with overlying mucosal necrosis and ulceration. They also vary in size, from a few mm to 40 cm in diameter. Many GISTs are well defined by a thin pseudocapsule. Over 95% of patients present with a solitary primary tumor, and in 10 to 40% of these cases the tumor directly invades neighboring organs.[6] Gastric GISTs are usually presented with GI bleeding and abdominal pain. However, most patients are asymptomatic and the lesions are discovered incidentally during an upper endoscopy performed for other reasons.[7] Their metastatic potential is difficult to predict due to the lack of clear clinical or pathological signs of malignancy other than obvious metastasis at surgery. In addition, local recurrence or distant metastasis may not present until years after the initial diagnosis.[8] Surgical resection is required to cure gastric GISTs. In the past, a 1 to 2 cm margin was thought to be necessary for an adequate resection.[7,9] Recently, DeMatteo *et al* demonstrated that tumor size and not negative microscopic surgical margins determine survival.[10] These finding support the local resection of GISTs lesions, including both wedge and gastric resections.

## MATERIALS AND METHODS

**Figure 1:**
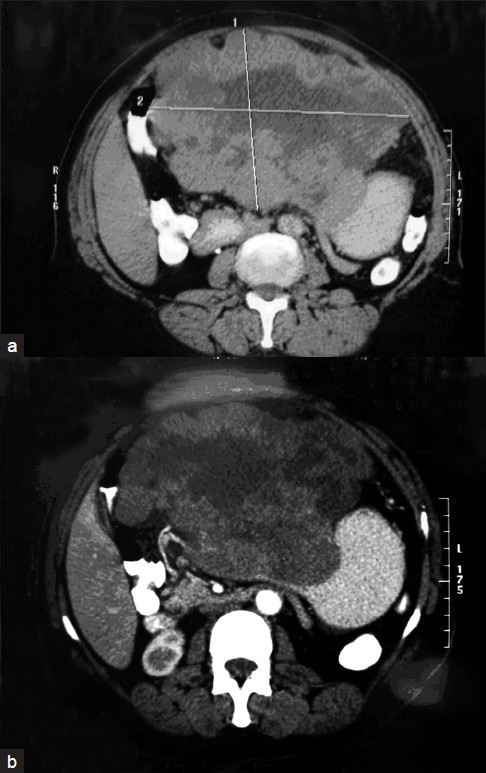
(a) Computed tomography (CT) for primary gastric gastrointestinal stromal tumors (GISTs); (b) CT for primary gastric GIST

From January 1997 to April 2008, thirty seven consecutive patients had undergone resection of GISTs in Gastro-entrology Center, Mansoura University, Egypt. They were distributed as gastric in 35 patients, one in the small intestine (terminal ileum), and one in the rectum (the two patients with intestinal GISTs were excluded from the study). All patients with gastric GISTs underwent upper endoscopy, whereas biopsies were done in 22 patients, barium meal in 19 patients and 26 patients had abdominal CT scan [Figure 1]. This raised the suspicion of GIST in 30 patients (85%). Patients with unresectable metastasis or concurrent cancer other than GIST were excluded. We started using the immunohistochemical analysis in 2002 in the remaining 24 patients and the CD 117 was positive in 22 patients (92%). Patient’s demographics and clinical presentation were analyzed. Perioperative parameters measured included operative times, estimated blood loss, intraoperative finding, surgical techniques, morbidity and length of hospitalization. Also recurrent disease and patients’ survival were analyzed.

### Postoperative care and follow-up

Postoperatively, nasogastric tubes were routinely used. A gastrograffin swallow was performed after the first week in pa0tients who underwent total gastrectomy. Diets were subsequently liberalized. Patients were discharged from hospital after developing tolerance to regular diet. In addition to routine visits at approximately 10 and 30 days after surgery, postoperative follow-up included physical examination every three to four months for the first two years, every six months for two years, and then yearly. A chest radiograph, abdominal computed tomography (CT) scan, and serum chemistries were obtained at six months, one year, and then annually for five years in the majority of patients. Upper endoscopy was performed at approximately six months and one year postoperatively and then repeated annually for two years. A CT scan, MR imaging, and/or chest CT scan were obtained if abnormalities were found on any of the surveillance studies.

## RESULTS

### Patients’ characteristics

From January 1997 to April 2008, thirty five consecutive patients who had undergone resection of gastric GIST were reviewed, of whom 37% were males and 63% were females. The median age was 59 ± 14 years (range, 23 to 75 years). The primary presenting symptoms are summarized in Table 1, where the most common symptoms were bleeding and dyspepsia. Anemia was present in 10 patients (29%), the mean hemoglobin level on admission was 6 ± 2.3gm%. Five patients (14%) were presented with weight loss (average 20% loss in the last three months). Table 2 shows the distribution of gastric lesions, where 13 were located in the fundus of the stomach, 15 in the body and seven in the pylorus. Tumor size was < 10 cm in the majority of patients (80%). The average tumor size was 6.3 ± 3.2cm (range from 3 to 13 cm). Table 3 shows the shape of the lesion: mass in 32 patients, ulcer in 2 patients and a polyp in one patient.

**Table 1:** Main presenting symptoms

Symptoms	No.(%) of patients (*N*=35)
GI bleeding	17 (49)
Anemia	10 (29
Dyspepsia	10 (29)
Weight loss	5 (14)
Mass	5 (14)

**Table 2:** Distributions of gastric lesions

Tumor location	No.(%) of patients
Fundus of the stomach	13 (37)
Gastric body	15 (43)
Pylorus of the stomach	7 (20)

**Table 3:** Shape of the lesion

Shape	No.(%) of patients
Mass	32 (91)
Ulcer	2 (6)
Polyp	1 (3)

### Surgical management

**Figure 2:**
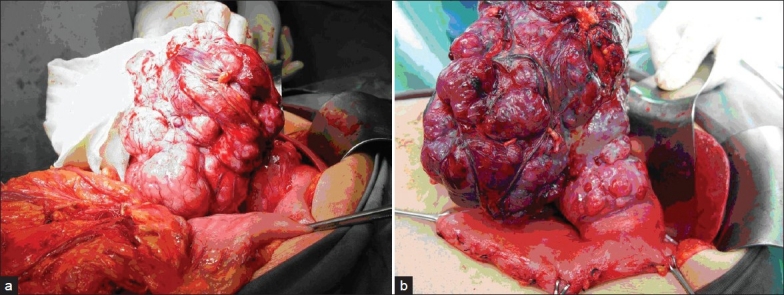
Operative photos showing large gastric GIST which was treated with wedge resection. (a) Dissection of gastric GIST; (b) Devascularization of stomach

The types of surgical resection are summarized in Table 4. A total of 20 (57%) patients underwent gastric wedge resection [Figure 2], eight (23%) patients underwent partial gastrectomy and the remaining seven patients (20%) underwent total gastrectomy.

**Table 4:** Type of surgical management

Type of gastrectomy	No.(%) of patients
Localized (Wedge)	20 (57)
Total gastrectomy	7 (20)
Subtotal gastrectomy	8 (23)

Combined adjacent organs resections were performed in 12 patients: spleen (nine patients), pancreas (distal pancreatectomy in two patients), gallbladder(one patient). Splenectomy was performed as a part of total gastrectomy in seven patients and in two patients with partial gastrectomy with enlarged splenic hilar lymph nodes. Distal pancreatectomy was performed in two patients when the tail of the pancreas was infiltrated by the tumor. Cholecystectomy was performed in one patient with concomitant gallbladder stone. Radical resections (Ro) were done for 32 patients (91.5%) while palliative resections were done for three patients (8.5%). The resected lymph nodes were negative in 32 patients (91.5%). The three- and five-years survival in patients who underwent wedge resection was 92% and 81% respectively, whereas it was 95% and 87% respectively in patients who underwent gastrectomy (either partial or total).

Recurrences were noted in three patients with a mean follow-up of 60 months. The median time of recurrence was 14.3 months (range, 7 to 28 months). We reoperated for recurrences in two patients. Only one patient remained disease free after complete excision for recurrent abdominal masses at gastric bed. The other patient had a peritoneal dissemination. The recurrence in the third patient occurred in the form of peritoneal seeding. These three patients were primarily having tumors ≥ 10cm.

The average operative time was 2.2 ± 1.7 h (range 1.5 to 4.5 h). The mean estimated blood transfusion was 1 unit (range 0 to 2 units). There were no major intraoperative complications. Post operatively, all patients required nasogastric tubes for 3 ± 1 days. No patients had any evidence of anastmotic leak, two patients had minor complications: obstructed gastro-jejunostomy where exploration and fixation of kinked loop was done and pancreatic leak which managed conservatively.

## DISCUSSION

GISTs may demonstrate a broad spectrum of biologic behavior from indolent to rapidly progressive malignancies. The identification of the precise cellular origin of such tumors has improved our knowledge of their natural history and malignant patients. Although surgery is the only way for curative therapy for these lesions, the preferred operative approach and extent of resection are still not well established. Our data demonstrates that surgical resection, either wedge resection or gastrectomy (partial or total), result in effective control of the disease with minimal perioperative morbidity and no mortality.

Most patients in our series were presented by symptoms of upper GI bleeding or had lesions discovered incidentally during a work-up of vague dyspeptic or reflux symptoms. Anemia or frank GI bleeding from an ulcerated tumor was encountered in 77% of patients. Following upper endoscopy, an abdominal CT scan is usually the test of choice to further delineate the location and size of the lesion and to look for direct or metastatic spread. Endoscopic ultrasound can be very helpful in diagnostic challenges.[11,12]

A demarcated hypoechoic mass that is contiguous with the muscularis propria layer of the stomach is characteristic of a GIST.[2,11] While endoscopic biopsies are frequently performed, they uncommonly yield anything more than normal mucosa.[2,11] In addition, a heterogeneous lesion larger than 4 cm, with irregular border, is reported to be highly suspicious for a malignant GIST.[13] However, if the images or pathology of the lesion being investigated do not show clear evidence of benign cyst or mass, it should be resected as a presumed GIST.

The stomach is by far the most common site of GISTs, occurring in 52 to 60% of the cases, with the proximal stomach involved in about two third of those patients.[11,14,15] In our study, the stomach represents the common site for GISTs (95%) and the middle third involved in 43% of those patients. Several studies described that most patients with gastric GIST are in the age group of 60-70 years, with only 10% of patients under 40 years of age.[8,14] In this series, six patients (17%) were under 40 years of age while 51% of the patients were in the age group of 60-70 years.

Surgical resection of localized gastric GISTs is the preferred treatment modality,[1,2,16] as resection of the tumor renders the only chance for cure at this time.[10,16] Historically 1-2 cm margin was thought to be necessary for an adequate resection.[7,9] However, more recently, DeMatteo *et al*,[10] demonstrated that tumor size and not negative microscopic surgical margins determine the survival. It is therefore accepted that the surgical goal should be a complete resection with gross negative margins only.[10,16] Given this, wedge resection has been advocated by many investigators for the majority of gastric GIST.[10,16–19] However, in some cases, tumor size and location may dictate a more extensive surgery, including partial or total gastrectomy.[14,20] In this study, 57% of the patients underwent a wedge resection and 43% underwent a gastrectomy (either partial or total). RO resections were done in 91% of patients. A combined adjacent organ resection was performed in 34% of all patients. In our study, the three and five-year survival in patients who underwent wedge resection was 92% and 81% respectively, whereas it was 95% and 87% respectively in patients who underwent gastrectomy (either partial or total). Our results are comparable to other series where Fujimoto *et al*, reported 93% 5-year survival[14] and Novitsky *et al*.[21] reported96% 3-year survival.

Although a simple resection appears feasible in most cases, the extent of surgery should be determined after considering the tumor size, location and relationship with adjacent organs to achieve a complete resection. However, the vital structure should not be sacrificed if the gross free margin has already been attained because the status of the microscopic margins does not appear to be important for survival.[10]

The recurrence rate after surgery in reported series ranged from 17% to 24%.[22–24] In our series, the recurrence rate was 8.5% with a median time of 14.3 months (range, 7 to 28 months). It was significantly lower than those reported in other series.[23,24] These three patients with recurrence primarily had tumors ≥ 10 cm, indicating that tumor size ≥ 10 cm carries a higher risk of recurrence. We also had a better five-year survival 84% compared with that reported in the literature (45% to 76%).[10,24–28] These superior patient results may have been caused by the fewer number of patients with tumor size ≥ 10cm (20%) and the principle of delicate surgical procedure to which we adhere. Another possible explanation is that during surgery, only one patient encountered tumor rupture which had been reported to be associated with early recurrence and poor prognosis. The predominant recurrence pattern of peritoneal seeding and the higher recurrence risk in patients with large tumor may be accounted for by the hypothesis that by the time tumor grows to a considerable size, it becomes more predisposed to peritoneal seeding by spreading out of the tumor by way of higher intratumor pressure or loosened tumor cellular adhesion, whereas smaller tumors are more likely to remain intact in the submucosal layer.

Although a complete surgical excision of tumors offer the best chance of a cure, high rates of metastasis and recurrence in high malignant potential GISTs highlights the need for effective non-surgical treatments.[29] Before the introduction of imatinib mesylate, no active treatment was available for patients with unresectable or metastasized malignant GISTs.[30,31] It inhibits signal transduction of the growth factor receptor c-KIT, which in turn inhibits cell proliferation and the metabolism. So it may improve the clinical outcomes of patients with incompletely resected, metastatic or recurrent gastric GIST.

In conclusion, complete surgical resection either through wedge resection or gastrectomy with negative margins remains the gold standard treatment in the management of patients with primary resectable gastric GIST.
